# Application of a Poisson deep neural network model for the prediction of count data in genome‐based prediction

**DOI:** 10.1002/tpg2.20118

**Published:** 2021-07-29

**Authors:** Osval A. Montesinos‐Lopez, Jose C. Montesinos‐Lopez, Eduardo Salazar, Jose Alberto Barron, Abelardo Montesinos‐Lopez, Raymundo Buenrostro‐Mariscal, Jose Crossa

**Affiliations:** ^1^ Facultad de Telemática Univ. de Colima Colima Colima 28040 México; ^2^ Dep. de Estadística Centro de Investigación en Matemáticas Guanajuato Guanajuato 36023 México; ^3^ Dep. of Animal Production (DPA) Universidad Nacional Agraria La Molina Av. La Molina, s/n La Molina 15024 Lima Perú; ^4^ Dep. de Matemáticas Centro Universitario de Ciencias Exactas e Ingenierías, Univ. de Guadalajara Guadalajara Jalisco 44430 México; ^5^ Biometrics and Statistics Unit International Maize and Wheat Improvement Center (CIMMYT) Carretera km 45, Mexico‐Veracruz Texcoco Edo. de México CP 52640 México; ^6^ Colegio de Post‐Graduados CP 56230, Montecillos, Edo. de México Texcoco México

## Abstract

Genomic selection (GS) is revolutionizing conventional ways of developing new plants and animals. However, because it is a predictive methodology, GS strongly depends on statistical and machine learning to perform these predictions. For continuous outcomes, more models are available for GS. Unfortunately, for count data outcomes, there are few efficient statistical machine learning models for large datasets or for datasets with fewer observations than independent variables. For this reason, in this paper, we applied the univariate version of the Poisson deep neural network (PDNN) proposed earlier for genomic predictions of count data. The model was implemented with (a) the negative log‐likelihood of Poisson distribution as the loss function, (b) the rectified linear activation unit as the activation function in hidden layers, and (c) the exponential activation function in the output layer. The advantage of the PDNN model is that it captures complex patterns in the data by implementing many nonlinear transformations in the hidden layers. Moreover, since it was implemented in Tensorflow as the back‐end, and in Keras as the front‐end, the model can be applied to moderate and large datasets, which is a significant advantage over previous GS models for count data. The PDNN model was compared with deep learning models with continuous outcomes, conventional generalized Poisson regression models, and conventional Bayesian regression methods. We found that the PDNN model outperformed the Bayesian regression and generalized Poisson regression methods in terms of prediction accuracy, although it was not better than the conventional deep neural network with continuous outcomes.

## Core Ideas

- Capturing patterns in the data by nonlinear transformations in the hidden layers is essential.
- The Poisson deep neural network (PDNN) is proposed for genomic predictions of count data.
- The PDNN method can be used with moderate and large datasets.
- The PDNN method captures signals in count data for genomic predictions.

**Abbreviations**
ASC: average Spearman correlation
BLRR: Bayesian log‐normal ridge regression
BNRR: Bayesian normal ridge regression
DNN: deep neuronal network
GPER: generalized Poisson elastic net regression
GPLR: generalized Poisson lasso regression
GPRR: generalized Poisson ridge regression
GS: genomic selection
MAAPE: mean arctangent absolute percentage error
MSE: mean square error of prediction
PDNN: Poisson deep neural network
PS: phenotypic selection
RELU: rectified linear activation unit.

## 1 INTRODUCTION

Selection is a key stage in crop breeding, and the conventional breeding process is based on phenotypic selection (PS). According to their experience, breeders choose appropriate offspring based on the observed phenotypes of crops in order to achieve the genetic improvement of target traits. However, conventional breeding strategies under PS are quite costly and time‐consuming. Genomic selection (GS), a predictive methodology for the selection of genotype candidates early in the breeding process, is revolutionizing plant breeding, as candidate genotypes can be selected without measuring the phenotypic information, which significantly reduces the time needed for the selection process (Crossa et al., 2017). This is possible because the selection process of individual candidates uses predicted values (breeding values or phenotypic values) from a statistical machine learning model, trained with a reference population that was genotyped and phenotyped. By statistical machine learning, for the purpose of this paper, we refer to conventional machine learning methods and statistical methods. According to the sum of all the marker effects, statistical machine learning prediction models tend to capture the total additive genetic variance to estimate the breeding values of individuals. However, to implement the GS methodology successfully, it is important to have a representative reference population that can be used to predict unseen phenotypes or even detect the breeding values of interest. It is equally important to have quality data for the genotypic and phenotypic information, as well as having good coverage of the markers of the complete genome; that is, the measured molecular markers should match the true objective population (the genome) (Battenfield et al., 2016; Crossa et al., 2017; Edwards et al., 2019; Guo et al., 2019). Additionally, it must be pointed out that selection of the statistical machine learning model is a key component for the successful application of GS (Montesinos‐López et al., 2019b).

The GS approach started in the animal breeding domain (Meuwissen et al., 2001) (mostly in dairy cattle), where a significant increase in its use has been observed (Zhang et al., 2015). This methodology has also been used in plant breeding for improving maize (*Zea mays* L.). For example, Vivek et al. (2017) compared GS and conventional PS and found that, for drought conditions, the gain per cycle under PS was 0.27, whereas under GS, it was 0.50. Under optimal conditions, the gain per cycle under PS was 0.34, but under GS, it was 0.55. The genetic gain per year for drought conditions under PS was 0.067 and under GS, it was 0.124; under optimal conditions, the gain per year was 0.084 for PS and 0.140 under GS. In contrast, Smallwood et al. (2019) reported that for yield, GS (calculated by BayesB and GBLUP) did not differ from PS (*P* > .05) in a soybean [*Glycine max* (L.) Merr.] population consisting of 276 F_5_ derived recombinant inbred lines. However, the traits of protein content, oil content, oleic acid, and linoleic acid chosen through the GS selection process (by BayesB and GBLUP) outperformed (*P* < .05) those chosen by PS (Smallwood et al., 2019). In the case of maize, Môro et al. (2019) reported that no significant differences were observed between the two, with PS being based on selection indices, rather than on each trait separately, in 256 plants of an F_2_ population (S_1_ lines) genotyped with 177 microsatellite molecular markers. In soybeans, Smallwood et al. (2019) found that for fatty acid traits, the genomic prediction method (with GBLUP and BayesB) outperformed PS, whereas for traits such as protein and oil, no significant differences were found between them. Although Salam and Smith (2016) reported similar gains from response to selection between GS and PS, they found some advantages, in that GS required a shorter breeding cycle and was more cost‐effective. These applications confirm that there are no relevant differences between GS and PS, and the advantages are that GS requires fewer resources because it reduces the cost per cycle and shortens the generation interval.

For these reasons, GS has been implemented in many crops such as maize and wheat (*Triticum aestivum* L.) (Crossa et al., 2017), chickpea (*Cicer arietinum* L.) (Roorkiwal et al., 2016), oil palm (*Elaeis guineensis* Jacq.) (Kwong et al., 2017), cassava (*Manihot esculenta* Crantz) (Wolfe et al., 2017), and rice (*Oryza sativa* L.) (Huang et al., 2019), among others. Nowadays, many breeding programs around the world are starting to move from conventional breeding programs (based on phenotyping) to GS (based on predictions of breeding values or phenotypic values), because there is increasing evidence that GS can significantly save resources and shorten the generation interval, as mentioned above (Crossa et al., 2017). However, some key issues still need to be addressed in order to implement GS more efficiently, such as improving the quality of the data (phenotypic and genotypic), the power of the statistical machine learning models, and the design of the training population size or composition, which, according to Tayeh et al. (2015), have a greater effect on the accuracy than the statistical machine learning model. With regard to the latter, it is important to point out that most of the time, algorithms that can deal with the problem of a large number of independent variables and a small number of observations are useful (i.e., algorithms that can work where the number of independent variables is larger than the number of observations). Some of the most popular ones are the Bayesian methods: Bayesian ridge regression, BayesA, BayesB, BayesC, Bayes Lasso, GBLUP, the RR‐BLUP method, etc. Some generalizations need to be taken into consideration when we use the Bayesian alphabet to model binary, ordinal and count traits via univariate and multivariate approaches. However, because there is no universal model that works well (outperforms) under all circumstances (Wolpert & Macready, 1997), many statistical machine learning models have been proposed and adapted for GS from other fields, some of which offer competitive prediction performance with regard to conventional genomic prediction models (Montesinos‐López et al., 2019a).

The most recently adopted models for GS are deep learning models (Bellot et al., 2018; Montesinos‐López, Montesinos‐López, Gianola, et al., 2018; Montesinos‐López, Montesinos‐López, Crossa, et al. 2018; Montesinos‐López et al., 2019a, 2019b). These models are generalizations of conventional artificial neural networks, since rather than working with only one hidden layer, they are able to incorporate many. These models are inspired by the functioning of the human brain, as they try to mimic how the brain works to perform a complex task. Furthermore, they have been implemented in many domains to create artificial intelligence devices and systems. For example, these methods have been used for Facebook's face recognition systems, other voice recognition systems, automatic book classification, self‐driving cars, the selection of human resources in big companies, and for skin cancer prediction (Chollet & Allaire, 2017), as well as for genomic prediction (Bellot et al., 2018; Montesinos‐López, Montesinos‐López, Gianola, et al., 2018; Montesinos‐López, Montesinos‐López, Crossa, et al. 2018; Montesinos‐López et al., 2019a, 2019b), among others. Some applications of deep learning for GS have recently been published for univariate and multivariate predictions (Bellot et al., 2018; Montesinos‐López, Montesinos‐López, Gianola, et al., 2018; Montesinos‐López, Montesinos‐López, Crossa, et al. 2018; Montesinos‐López et al., 2019a, 2019b), showing that these models provide predictions that are very competitive with conventional statistical machine learning models, especially in cases where larger datasets are available (Chollet & Allaire, 2017), and better prediction performance can be achieved. These applications have been used for predicting continuous, binary, count (Montesinos‐López et al., 2020), and ordinal traits under univariate and multivariate frameworks.

**Core Ideas**

- Capturing patterns in the data by nonlinear transformations in the hidden layers is essential.
- The Poisson deep neural network (PDNN) is proposed for genomic predictions of count data.
- The PDNN method can be used with moderate and large datasets.
- The PDNN method captures signals in count data for genomic predictions.

Count data reflect the number of occurrences of an outcome variable measured in a fixed period of time (for example, per hour or day), area, or volume. Usually, the Poisson family of regressions, which assume that the variance is equal to the mean, is used to model count data (Stroup, 2012). In medicine and health care, these count data are collected every hour or day and may consist of laboratory test results, adverse drugs events, etc. (Du et al., 2011). Likewise, statisticians, econometricians and social scientists have also studied modeling for count data. Count data are common in plant breeding and can be used to find the panicle number per plant, number of seeds per plant, and number of infected spikelets per plant, days to heading, and days to maturity, among others (Montesinos‐López et al., 2016; Montesinos‐López et al., 2017). When count data are used, the dependent variable takes on values of 0, 1, 2…, with an unrestricted upper limit.

The classic method of estimation under the maximum likelihood of Poisson regression models is not efficient for large datasets. For this reason, other alternatives have been explored, such as Bayesian methods that use the Gibbs sampler method, which is powerful when generating samples from high‐dimensional probability distributions; these became even more efficient when closed‐form Gibbs samplers were developed (Park & van Dyk, 2009). However, closed‐form Gibbs samplers have been developed for Bayesian Poisson regression and have been inefficient. Three publications on the implementation of Bayesian Poisson regression for genomic predictions were published by Montesinos‐López et al. (2015), Montesinos‐López et al. (2016), and Montesinos‐López et al. (2017). However, because these methods are based on a complex Gibbs sampler, which uses an inefficient mixing process to generate samples from high dimensional probability distributions, the implementation of these three methods is difficult with moderate or large datasets.

On the basis of the previous considerations, Montesinos‐López et al. (2020) proposed a deep learning genomic prediction model for count data that can be implemented with large datasets for genomic prediction. Deep neural network models are not new in GS, as there are applications for continuous, binary count and ordinal outcomes that show that these models are very competitive and are sometimes better than conventional statistical models (Montesinos‐López, Montesinos‐López, Gianola, et al., 2018; Montesinos‐López, Montesinos‐López, Crossa, et al. 2018; Montesinos‐López et al., 2019a, 2019b; Montesinos‐López, et al., 2020). It is important to point out that some authors, such as Fallah et al. (2009) and Rodrigo and Tsokos (2019), proposed nonlinear Poisson regression models for artificial neural networks; however, these models were implemented via a classic and Bayesian approaches, making them quite inefficient for large datasets. Moreover, these models were developed with only one hidden layer and, as such, belong to conventional artificial neural network models and not to deep learning models. Yet another disadvantage of these models is that the authors did not provide software for their user‐friendly implementation.

On the other hand, the method proposed by Montesinos‐López et al., (2020) allows more than one hidden layer; for this reason, it belongs to the deep neural network family for count data. For this reason, in this paper, the Poisson deep neural network (PDNN) model for count data will be implemented and benchmarked with other conventional methods (Bayesian methods for continuous outcomes, generalized Poisson regression, and deep learning for continuous outcomes). The PDNN model needs to be trained with the negative log‐likelihood of a Poisson distribution as the loss (or objective) function, but with the exponential function as the activation function for the output layer in order to guarantee positive outcomes. Moreover, the PDNN model is trained under the back‐propagation algorithm, which is considered the king algorithm for training deep learning models. Because they transform the original input, deep learning models capture complex nonlinear patterns by applying nonlinear transformations to the input and output of the neurons of each hidden layer. Additionally, by increasing the number of hidden layers, they are able to capture the more complex nonlinear patterns of the input information. The back‐propagation method involves the numerical evaluation of derivatives of the error function to update the weights and biases. Furthermore, to implement this PDNN model more efficiently for large datasets, we will use Tensorflow as the back‐end and Keras as the front‐end in R (Chollet & Allaire, 2017).

## 2 MATERIAL AND METHODS

### 2.1 Generalized Poisson regression model

According to Montesinos‐López et al. (2020), the Poisson distribution with a parameter μ belongs to the exponential family, and its probability function is:

(1) $$\begin{array}{ccc} f\;\left(y_{i}\right) & = & \frac{e^{-\mu_{i}}\mu_{i}^{y_{i}}}{y_{i}!}\\ y_{i} & = & \;0,\;1,\;2,\cdots , \end{array}$$

where $y_{i}\;$= 0,1,2,3,… is the value of the counting variable associated with unit i, given a set of explanatory variables. The mean and variance of a Poisson random variable are $E\;\left(y_{i}\right)=\;Var\;\left(y_{i}\right)=\mu_{i}\;$. This Poisson distribution is often used to model responses that are “counts”. Given that our training set is composed of pairs of inputs ($y_{i}$, $x_{i}^{T}$) with $x_{i}^{T}=\;\left[x_{i1},\;\ldots ,x_{ip}\right]$, for i=1,2,…,n, the logarithm of the likelihood is given by:

(2) $$\begin{array}{ccc} logf\;\left(y_{i}\right) & = & \;log\;\left(\prod_{i\;=\;1}^{n}\frac{e^{-\mu_{i}}\mu_{i}^{y_{i}}}{y_{i}!}\right)\\ & = & \sum_{i=1}^{n}\left[-\mu_{i}+y_{i}log\left(\mu_{i}\right)-log\left(y_{i}!\right)\right]. \end{array}$$

The specification of a generalized Poisson regression model is given as predictor = $log\left(\mu_{i}\right)=\eta +\Sigma_{\left(j=1\right)}^{p}\;x_{ij}\beta_{j}$, distribution = $y_{i}\;∼\;Poisson\left(\mu_{i}\right)$, and link function = log, where η is the intercept; $x_{ij}$ is the *j* ^th^ independent variable measured in observation i, where j=1,2,..,p; and $\beta_{j}$ is the beta coefficient corresponding to the independent variable j. Thus, the expected value is $E\;\left(y_{i}x_{i}^{T}\right)=\mu_{i}\;=\;\exp\left(\eta +\sum_{j\;=\;1}^{p}x_{ij}\beta_{j}\right)$. Since the link function is the log function, this means that the *inverse link function* is the exponential function, which is called the *activation function* in the specification of the PDNN model. The optimization process can be performed by minimizing the negative log‐likelihood (called the loss function); however, when the number of independent variables (p) is larger than the number of observations (*n*), we suggest using a penalized version of the negative log‐likelihood (LL) which is equal to:

(3) $$\begin{array}{ccc} LL & = & -\;\sum_{\left(i=1\right)}^{n}\left[-\mu_{i}+y_{i}log\left(\mu_{i}\right)\right]\\ & & +\;\lambda \left(\left(1-\alpha \right)\left[\sum_{j\;=\;1}^{p}\beta_{j}^{2}+\alpha \sum_{j\;=\;1}^{p}\left|\beta_{j}\right|\right]\right), \end{array}$$

where λ is the tuning hyperparameter that can be chosen by cross‐validation and α is a parameter that causes Ridge penalization, Lasso penalization, or a mixture of both. For example, when α=0, the log‐likelihood corresponds to a generalized Poisson Ridge regression (GPRR); when α=1, the log‐likelihood corresponds to a generalized Poisson Lasso regression (GPLR); and when when 0<α<1, the log‐likelihood corresponds to a generalized Poisson elastic net regression (GPER). The optimization of this loss function (LL) was carried out by the R package glmnet (lasso and elastic‐net regularized generalized linear models) (Friedman et al., 2010). The selection of the tuning hyperparameter (λ) was performed through 10‐fold cross‐validations created with each training set. By default, the library generates 100 values of λ to make up the grid; each one of them was then evaluated under an inner 10‐fold cross‐validation and the best value of λ was selected. Next, with the optimal value of λ, the GPRM was refitted and, in this final trained model, the prediction performance was computed on each of the folds of the testing set.

### 2.2 Poisson deep neural network model

**FIGURE 1:**
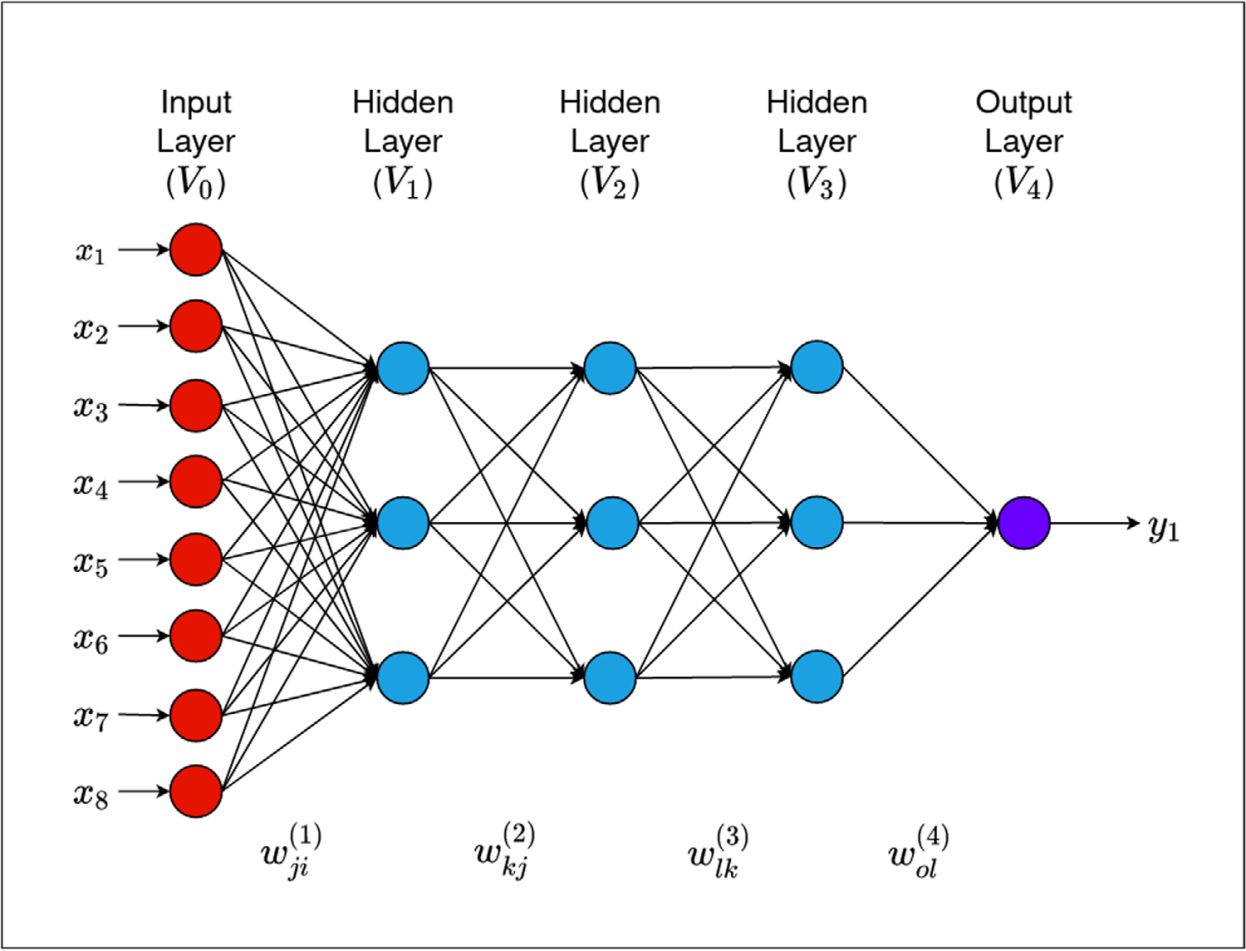
A feed‐forward deep neural network with one input layer, three hidden layers, and one output layer. There are eight neurons in the input layer that correspond to the input information and three neurons in each of three hidden layers, with only one neuron in the output layer that corresponds to the count trait that will be predicted (intercepts = biases not shown in this figure)

In this study, we implemented a simple feed‐forward neural network that is also called a multilayer perceptron, as shown in Figure 1. The topology of deep neural networks consists of an input layer (8 inputs, as shown in Figure 1), one output layer (see Figure 1) and several hidden layers (3, as shown in Figure 1). The input is passed on to the neurons in the first hidden layer, and then each hidden neuron produces an output that is used as an input for each of the neurons of the second hidden layer. Similarly, the output of each neuron in the second hidden layer is used as an input for each neuron in the third hidden layer. Finally, the output of each neuron in the third hidden layer is used as an input to obtain the predicted values of the response variable of interest. It is important to point out that in each of the hidden layers, we obtained a weighted sum of the inputs and weights (including the intercept), which is called the net input, to which a transformation called the activation function is applied to produce the output of each hidden neuron (Montesinos‐López et. al., 2020).

The analytical formulas of the model given in Figure 1 for *one* output, with d inputs (not only 8), $M_{1}$ hidden neurons (units) in hidden layer 1, $M_{2}$ hidden units in hidden layer 2, $M_{3}$ hidden units in hidden layer 3, and one output neuron are given by the following Equations ((1), (2), (3), (4)):

(4) $$\begin{array}{ccc} V_{1j} & = & g_{1}\left(\Sigma_{\left(i=1\right)}^{d}w_{\left(\mathrm{ji}\right)}^{\left(1\right)}x_{\left(i\right)}+{b_{j}}_{1}\right)\\ & & \mathrm{for}\;\;j=1,\;\;\cdots \;,M_{1} \end{array}$$

(5) $$\begin{array}{ccc} V_{2k} & = & g_{2}\left(\Sigma_{\left(j=1\right)}^{\left(M_{1}\right)}{w_{\mathrm{kj}}}^{\left(2\right)}V_{1j}+b_{k2}\right)\\ & & \mathrm{for}\;\;k=1,\;\;\cdots \;,M_{2} \end{array}$$

(6) $$\begin{array}{ccc} V_{3l} & = & g_{3}\left(\Sigma_{\left(k=1\right)}^{\left(M_{2}\right)}{w_{\mathrm{lk}}}^{\left(3\right)}V_{2k}+b_{l3}\right)\\ & & \mathrm{for}\;\;l=1,\;\;\cdots \;,M_{3} \end{array}$$

(7) $$\;y\;=g_{4}\;\left(\sum_{l\;=\;1}^{3}w_{l}^{\left(4\right)}V_{3l}+b_{4}\right)$$

where $g_{1},\;g_{2},\;g_{3}$, and $g_{4}$ are activation functions for the first, second, third, and output layers; $M_{1}$, $M_{2}$, and $M_{3}$ are the required number of neurons in each layer, respectively, and the values used for these neurons are provided later in the grid. Equation (4) produces the output of each of the neurons in the first hidden layer, Equation (5) produces the output of each of the neurons in the second hidden layer, Equation (6) produces the output of each of the neurons in the third hidden layer and, finally, Equation (7) produces the output of the unique response variable of interest. The learning process involves updating the weights 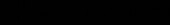 and biases $\left(b_{j1},\;b_{k2},\;b_{l3},\;b_{4}\right)$ to minimize the loss function, where these weights and biases correspond to the first hidden layer $\left(w_{\left(ji\right)}^{\left(1\right)},b_{j1}\right)$, the second hidden layer $\left({w_{kj}}^{\left(2\right)},b_{k2}\right)$, the third hidden layer $\left({w_{lk}}^{\left(3\right)},b_{l3}\right)$, and the output layer $\left({w_{ol}}^{\left(4\right)},b_{4}\right)$, respectively. To obtain the outputs of each of the neurons in the three hidden layers ($g_{1},\;g_{2},\;\mathrm{and}\;g_{3}),\;$we used the rectified linear activation unit (RELU). However, for the output layer, we used the exponential activation functions ($g_{4})$ because the response variables that we wanted to predict are counts. According to the universal approximation theorem, a neural network with enough hidden units can approximate any arbitrary functional relationship (Cybenko, 1989; Hornik, 1991). The PDNN model was implemented with the Keras library as the front‐end, Tensorflow as the back‐end (Chollet & Allaire, 2017), and the Adaptive Moment Estimation (Adam) as the optimizer. It is important to point out that the implemented PDNN model is really a deep learning model when the number of hidden layers is more than one; however, to avoid giving two names to the proposed model, we only used the term PDNN, with the understanding that when one hidden layer is used, this is a conventional artificial neural network. The PDNN is therefore the univariate version of the multivariate deep neural network proposed by Montesinos‐López et al. (2020).

To select the hyperparameters, we performed a grid search with 20 combinations: 10 values for the number of neurons per layer (123, 148, 173, 198, 223, 248, 273, 298, 323, and 348) and two values for the λ parameters for the lasso penalization (0.001 and 0.01). The remaining hyperparameters were fixed. The number of epochs used was 1,000, the dropout percentage was set to zero, the batch size was 320, the validation split was 20% of the outer training set, the learning rate was equal to 0.001, and the activation function for the hidden layers was RELU. It is important to point out that each of the 20 combinations was run with these fixed hyperparameters with one, two, three, and four hidden layers, via the early stopping approach that allows the selection of the optimal number of epochs and with the minimum of the Poisson loss score as the criterion (for the stopping rule). This means that the 10 number units mentioned above were run with each of the hidden layers and that the number of neurons in the first, second, and third hidden layers was the same. From these 20 combinations, we selected the best combination for each hidden layer (one, two, three, and four) in terms of the lower mean square error of prediction inside each outer training set. With this optimal combination of hyperparameters, we refitted the model with all the information of the outer training set. Finally, the predictions were made for the corresponding outer testing set by using the estimates of the refitted model with the optimal hyper‐parameters. This process was carried out in each of the five folds. Since the optimal hyperparameters were selected in each hidden layer (one, two, three, and four), the four models were evaluated under the PDNN. We also ran the normal counterpart of the PDNN, which we named the deep neuronal network (DNN), under the same topology and hyperparameter settings. The only difference in the implementation of the normal DNN was that instead of using the Poisson loss function, we used the mean square error loss function, which is appropriate for continuous outcomes. Moreover, since the generalized Poisson regression model depends on the value of α, five models were developed by changing the value of α. Table 1 shows the 15 models that we generated. Under the two previous models (the generalized Poisson regression model and PDNN model) as a predictor, we included not only the information on markers but also the information on environments and the genotypes × environment interaction term. The information on environments was added by creating the design matrix of the environments, and the information on genotypes was created by building the design matrix of the genotypes that was later multiplied by the square root of the genomic relationship matrix, whereas the genotype × environment interaction term was built from the design matrix of environments and the design matrix of genotypes that took the genomic relationship matrix as input.

**TABLE 1:** Proposed and implemented models

Model	Model name	Abbreviation of model	Hidden layer	Alpha^a^
1	Normal deep neural network	DNN_1	1	NN
2	Normal deep neural network	DNN_2	2	NN
3	Normal deep neural network	DNN_3	3	NN
4	Normal deep neural network	DNN_4	4	NN
5	Poisson deep neural network	PDNN_1	1	NN
6	Poisson deep neural network	PDNN_2	2	NN
7	Poisson deep neural network	PDNN_3	3	NN
8	Poisson deep neural network	PDNN_4	4	NN
9	Generalized Poisson Ridge regression	GPRR	0	0
10	Generalized Poisson Lasso regression	GPLR	0	1
11	Generalized Poisson Elastic net regression	GPER_.25	0	.25
12	Generalized Poisson Elastic net regression	GPER_.5	0	.5
13	Generalized Poisson Elastic net regression	GPER_.75	0	.75
14	Bayesian normal Ridge regression	BNRR	0	NN
15	Bayesian log normal Ridge regression	BLRR	0	NN

^a^ Alpha is the parameter α and is needed only in generalized Poisson regression models. ^b^ NN, this parameter was not needed for the model.

### 2.3 Bayesian models

Note that the Bayesian normal Ridge regression (BNRR) and the Bayesian log‐normal Ridge regression (BLRR) models were implemented in the BGLR package of de los Campos and Pérez‐Rodríguez (2014), assuming a normal response variable under a Bayesian framework with the weakly informative priors described in the package. To implement the BLRR model in the BGLR package, the original count response variable was transformed as log(count+0.1) before its implementation. After the predicted values had been obtained, they were transformed back to the original count by the exponential (predicted) values −0.1 to guarantee that the comparisons were on the same scale. This means that the BLRR is not a true Bayesian log‐normal model, since we only used an approximation by implementing the transformation above.

### 2.4 Data

#### 2.4.1 Phenotypic data

The phenotypic dataset included 115 spring wheat lines developed by the International Maize and Wheat Improvement Center that were assembled and evaluated for resistance to *Fusarium graminearum* at the El Batan experimental station in Mexico in 2011 over the course of three experiments. For the application, we called these three experiments Env1, Env2, and Env3. In all the experiments (environments), the genotypes were arranged in a randomized complete block design, in which each plot comprised two 1‐m double rows separated by a 0.25‐m space. *Fusarium* head blight severity data were collected 20 and 30 d before maturity by counting symptomatic spikelets on five randomly selected spikes in each plot. We used the counts collected after 20 d and a dataset containing 6,480 observations. These datasets were the same ones that Montesinos‐López et al. (2016) used in their paper for count data with genotype× environment interactions. It should be pointed out that this dataset is different to the one used by Montesinos‐López et al. (2020), although the experiments were conducted in the same place.

#### 2.4.2 Genotypic data

DNA samples were extracted from young, 2‐ to 3‐wk old leaves, taken from each line, via Wizard Genomic DNA purification (Promega), following the manufacturer's protocol. DNA samples were genotyped by an Illumina 9K SNP chip with 8,632 single nucleotide polymorphisms (Cavanagh et al., 2013). For a given marker, the genotype for the *i* ^th^ line was coded as the number of copies of a designated marker‐specific allele carried by the *i* ^th^ line (absence = 0, presence = 1). Single nucleotide polymorphisms markers with unexpected AB (heterozygous) genotypes were recoded as either AA or BB, according to the graphical interface visualization tool of GenomeStudio (Illumina) software. In addition, 66 simple sequence repeat markers were screened. After filtering the markers for 0.05 minor allele frequencies and deleting markers with 10% of no calls, the final set of single nucleotide polymorphisms included 1,635 single nucleotide polymorphisms. Genotypic and phenotypic information is given in further detail in Table 2.

**TABLE 2:** Description of the dataset

Item	Number	Details
Total observations	6,480	Divided into three environments and 115 lines
Environments	3	Batan2012 with 2,196 observations, Batan2014 with 2,197 observations, and Chunchi2014 with 2,087 observations
Lines	115	Average observations per line: 56.348; average observations per line in the Batan2012 environment: 19.095; average observations per line in the Batan2014 environment: 19.104; average observations per line in the Chunchi2014 environment: 18.147
Markers	1,635	Coded as the number of copies of a designated marker‐specific allele carried by the *i* ^th^ line (absence = 0; presence = 1)

### 2.5 Data repository

These datasets were the same ones used by Montesinos‐López et al. (2016) in their paper on count data with genotype× environment interactions. The phenotypic and genotypic data used in this study are contained in the R file Data_Real_Count.RData, and available at http://hdl.handle.net/11529/10575 (accessed 14 June 2021). This link is the same as the one given in Montesinos‐López et al. (2016) under the title ‘Genomic Bayesian Prediction Model for Count Data with Genotype × Environment Interaction’.

### 2.6 Metrics to measure prediction performance

Our study used cross‐validation, as this is used for model selection, as well as for evaluating the prediction performance in unseen data. Since our data contain the same lines in each of the *i* = 3 environments, we used a type of cross‐validation that mimicked a situation where the lines were evaluated in some environments for all traits but where some lines were missing in other environments. We implemented a five‐fold cross‐validation, where four folds were used for training and one for testing, to guarantee that we did not use the testing set for training the algorithms, thus avoiding overoptimistic predictions. We reported the average prediction performance for the test data in terms of the average Spearman correlation (ASC), mean square error of prediction (MSE), and mean arctangent absolute percentage error (MAAPE) for each environment.

Part of the training set was used for validation (inner testing) to be able to tune the hyperparameters. The hyperparameter (λ) in the Poisson regression was tuned with 10‐fold cross‐validation; that is, 10% of each training set was used for tuning (inner testing) and the remaining portion for training (inner training). However, the tuning process for hyperparameter selection from the 20 combinations of hyperparameters in the grid search under the PDNN model was carried out with 20% of the training set for validation (inner testing) and the remaining 80% for training (inner training). After selecting the best combination of hyperparameters in each fold, the model was refitted with this optimal set of hyperparameters; for its corresponding testing sets, the prediction performance was evaluated with ASC, MSE, and MAAPE, and the average of each metric of the five folds was reported as the metric of prediction performance. Moreover, the SE across the five folds was reported for each metric, meaning that the average and SE were calculated with five values for each metric (ASC, MSE, and MAAPE), one from each fold. It is important to point out that the fivefold cross‐validation strategy was implemented with only one replication.

## 3 RESULTS

**FIGURE 2:**
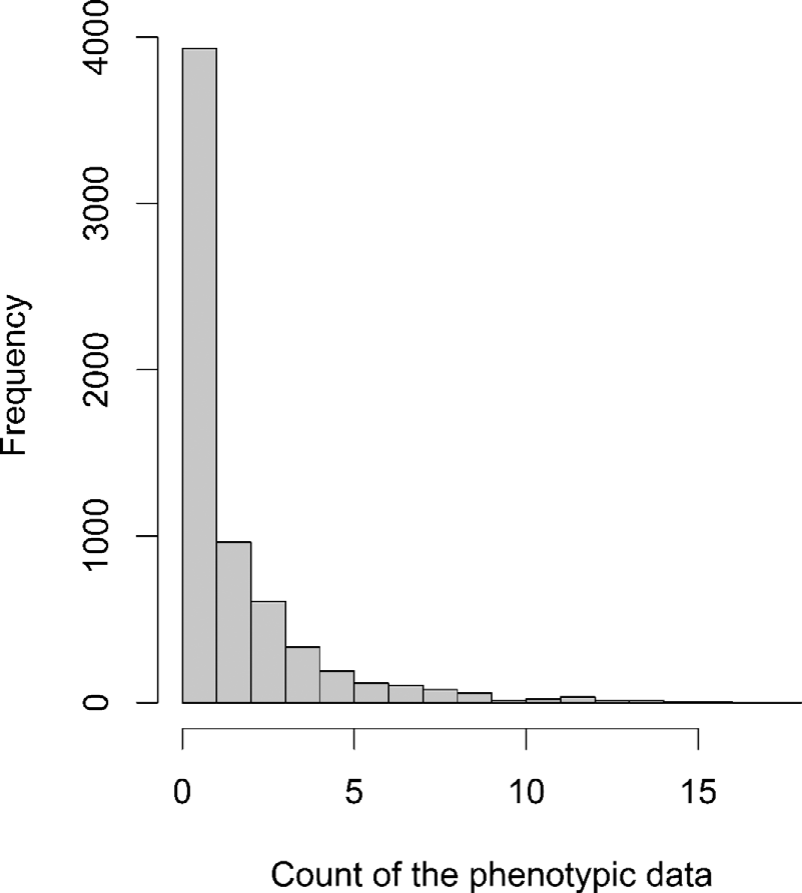
Distribution of the count response variable

The results are given in three sections. The first section provides the results of the 15 models, ignoring the genotype×environment interaction. The second provides the results that consider the genotype × environment interaction, whereas the last section gives a summary of the best model across environments. Before going to the first section of the results, it is worth pointing out that Figure 2 clearly shows that the phenotypic count data are not normally distributed and, for this reason, Poisson distribution was considered a better alternative.

### 3.1 Prediction performance ignoring the genotype×environment interaction

First, we compared the prediction performance of the 15 models across environments and then in each environment. The model abreviations are given in Table 1. Table 3 shows that the best predictions across environments were under the DNN_1 model with ASC = 0.656 and MSE = 1.346, and under the PDNN_1 model with MAAPE = 0.801. The worst prediction regarding ASC (ASC = 0.413) was observed under the BNRR model, whereas the worst prediction in terms of MSE and MAAPE was observed under the BLRR model with MSE = 5.070 and MAAPE = 0.871, respectively. The best model (DNN_1) outperformed the worst model by (0.656 – 0.413) ×100/0.413 = 59.047% and (5.070 – 1.807) ×100/1.807 = 180.50% in terms of ASC and MSE, respectively. The best model (PDNN_1) outperformed the worst model by 8.743% in terms of MAAPE.

**TABLE 3:** Prediction performance in terms of average Spearman correlation (ASC), mean square error (MSE), and mean arctangent absolute percentage error (MAAPE), ignoring the genotype×environment (WI) interaction of the 15 models; averages of each environment are indicated in bold

Model	Model name^a^	Without interaction	Environment	ASC	SE_1^b^	MSE	SE_2	MAAPE	SE_3
1	DNN_1	WI	Batan2012	0.576	0.011	1.317	0.034	0.896	0.012
1	DNN_1	WI	Batan2014	0.584	0.025	1.308	0.050	0.897	0.012
1	DNN_1	WI	Chunchi2014	0.809	0.003	2.797	0.151	0.623	0.012
1	DNN_1	WI	**Average**	**0.656**	**0.013**	**1.807**	**0.078**	**0.805**	**0.012**
2	DNN_2	WI	Batan2012	0.531	0.011	1.454	0.041	0.911	0.012
2	DNN_2	WI	Batan2014	0.536	0.027	1.448	0.054	0.912	0.012
2	DNN_2	WI	Chunchi2014	0.813	0.001	2.748	0.148	0.623	0.011
2	DNN_2	WI	**Average**	**0.627**	**0.013**	**1.883**	**0.081**	**0.815**	**0.012**
3	DNN_3	WI	Batan2012	0.533	0.010	1.449	0.040	0.910	0.012
3	DNN_3	WI	Batan2014	0.538	0.025	1.446	0.056	0.911	0.011
3	DNN_3	WI	Chunchi2014	0.813	0.001	2.766	0.169	0.623	0.012
3	DNN_3	WI	**Average**	**0.628**	**0.012**	**1.887**	**0.088**	**0.815**	**0.012**
4	DNN_4	WI	Batan2012	0.531	0.011	1.463	0.042	0.912	0.012
4	DNN_4	WI	Batan2014	0.537	0.025	1.455	0.056	0.911	0.013
4	DNN_4	WI	Chunchi2014	0.814	0.001	2.810	0.169	0.623	0.013
4	DNN_4	WI	**Average**	**0.627**	**0.012**	**1.909**	**0.089**	**0.815**	**0.013**
5	PDNN_1	WI	Batan2012	0.555	0.020	1.384	0.070	0.888	0.011
5	PDNN_1	WI	Batan2014	0.561	0.010	1.381	0.048	0.887	0.011
5	PDNN_1	WI	Chunchi2014	0.773	0.014	3.368	0.371	0.627	0.014
5	PDNN_1	WI	**Average**	**0.630**	**0.015**	**2.044**	**0.163**	**0.801**	**0.012**
6	PDNN_2	WI	Batan2012	0.366	0.003	2.183	0.157	0.903	0.013
6	PDNN_2	WI	Batan2014	0.354	0.005	2.191	0.150	0.904	0.008
6	PDNN_2	WI	Chunchi2014	0.636	0.009	9.617	1.611	0.714	0.016
6	PDNN_2	WI	**Average**	**0.452**	**0.006**	**4.664**	**0.639**	**0.840**	**0.012**
7	PDNN_3	WI	Batan2012	0.531	0.012	1.452	0.044	0.909	0.012
7	PDNN_3	WI	Batan2014	0.538	0.026	1.448	0.057	0.908	0.012
7	PDNN_3	WI	Chunchi2014	0.813	0.002	2.757	0.165	0.623	0.011
7	PDNN_3	WI	**Average**	**0.627**	**0.013**	**1.886**	**0.089**	**0.813**	**0.012**
8	PDNN_4	WI	Batan2012	0.545	0.013	1.604	0.208	0.899	0.008
8	PDNN_4	WI	Batan2014	0.560	0.032	1.629	0.225	0.898	0.016
8	PDNN_4	WI	Chunchi2014	0.778	0.016	5.226	1.832	0.655	0.024
8	PDNN_4	WI	**Average**	**0.628**	**0.020**	**2.820**	**0.755**	**0.817**	**0.016**
9	GPRR	WI	Batan2012	0.346	0.015	1.909	0.075	0.897	0.009
9	GPRR	WI	Batan2014	0.347	0.029	1.901	0.067	0.896	0.013
9	GPRR	WI	Chunchi2014	0.598	0.010	6.467	0.417	0.709	0.010
9	GPRR	WI	**Average**	**0.430**	**0.018**	**3.426**	**0.186**	**0.834**	**0.011**
10	GPLR	WI	Batan2012	0.347	0.014	1.912	0.075	0.900	0.009
10	GPLR	WI	Batan2014	0.347	0.029	1.906	0.068	0.900	0.013
10	GPLR	WI	Chunchi2014	0.598	0.010	6.431	0.414	0.712	0.011
10	GPLR	WI	**Average**	**0.431**	**0.018**	**3.416**	**0.186**	**0.837**	**0.011**
11	GPER_.25	WI	Batan2012	0.347	0.015	1.912	0.076	0.901	0.009
11	GPER_.25	WI	Batan2014	0.347	0.029	1.906	0.068	0.900	0.013
11	GPER_.25	WI	Chunchi2014	0.598	0.010	6.431	0.414	0.712	0.011
11	GPER_.25	WI	**Average**	**0.431**	**0.018**	**3.416**	**0.186**	**0.838**	**0.011**
12	GPER_.5	WI	Batan2012	0.347	0.014	1.912	0.076	0.901	0.009
12	GPER_.5	WI	Batan2014	0.347	0.029	1.906	0.068	0.900	0.013
12	GPER_.5	WI	Chunchi2014	0.598	0.010	6.432	0.414	0.712	0.011
12	GPER_.5	WI	**Average**	**0.431**	**0.018**	**3.417**	**0.186**	**0.838**	**0.011**
13	GPER_.75	WI	Batan2012	0.347	0.014	1.912	0.075	0.901	0.009
13	GPER_.75	WI	Batan2014	0.347	0.029	1.906	0.068	0.900	0.013
13	GPER_.75	WI	Chunchi2014	0.597	0.010	6.432	0.414	0.712	0.011
13	GPER_.75	WI	**Average**	**0.430**	**0.018**	**3.417**	**0.186**	**0.838**	**0.011**
14	BNRR	WI	Batan2012	0.339	0.015	2.098	0.088	0.930	0.010
14	BNRR	WI	Batan2014	0.342	0.029	2.093	0.069	0.930	0.012
14	BNRR	WI	Chunchi2014	0.557	0.014	7.486	0.434	0.738	0.008
14	BNRR	WI	**Average**	**0.413**	**0.019**	**3.892**	**0.197**	**0.866**	**0.010**
15	BLRR	WI	Batan2012	0.472	0.012	2.095	0.076	0.947	0.007
15	BLRR	WI	Batan2014	0.473	0.026	2.102	0.053	0.948	0.009
15	BLRR	WI	Chunchi2014	0.433	0.012	11.014	0.782	0.717	0.010
15	BLRR	WI	**Average**	**0.459**	**0.017**	**5.070**	**0.304**	**0.871**	**0.009**

^a^ DNN_1, normal deep neural network with one hidden layer; DNN_2, normal deep neural network with two hidden layers; DNN_3, normal deep neural network with three hidden layers; DNN_4, normal deep neural network with four hidden layers; PDNN_1, Poisson deep neural network with one hidden layer; PDNN_2, Poisson deep neural network with two hidden layers, PDNN_3, Poisson deep neural network with three hidden layers; PDNN_4, Poisson deep neural network with four hidden layers; GPRR, generalized Poisson Ridge regression; GPLR, generalized Poisson Lasso regression; GPER_0.25, generalized Poisson elastic net regression with α = .25; GPER_2, generalized Poisson elastic net regression with α = .5; GPER_.75, generalized Poisson elastic net regression with α = .75; BNRR, Bayseian normal Ridge regression; BLRR, Bayesian log‐normal ridge regression.

^b^ SE_1, SE of ASC; SE_2, SE of MSE; SE_3, SE of MAAPE.

We then compared the prediction performance in each environment. For the environment Batan2012 in terms of ASC and MSE, the best predictions were obtained under the DNN_1 model with ASC = 0.576 and MSE = 1.317, and under the PDNN_1 model, with MAAPE = 0.888. The worst prediction regarding ASC was under the BNRR model with ASC = 0.339, whereas in terms of MSE, it was observed under the PDNN_2 model with MSE = 2.183; for MAAPE, it was observed under the BLRR model with MAAPE = 0.947. The best models were DNN_1, DNN_1, and PDNN_1 in terms of ASC, MSE and MAAPE, respectively, and outperformed the worst model by 69.912% for ASC, by 65.760% for MSE, and by 6.644% for MAAPE (Table 3). In the environment Batan2014 in terms of ASC, MSE, and MAAPE, the best predictions were obtained with the models DNN_1 (ASC = 0.584), DNN_1 (MSE = 1.308), and PDNN_1 (MAAPE = 0.887), respectively, and the worst with the BNRR (ASC = 0.342), PDNN_2 (MSE = 2.191), and BLRR (MAAPE = 0.948), respectively. Regarding ASC, the best model outperformed the worst model by 70.760%, whereas for MSE, the worst model was outperformed by 67.510%, and, for MAAPE, the best model outperformed the worst by 6.877% (Table 3). Finally, with regard to the environment Chunchi2014 in terms of ASC, the best and worst predictions were observed in DNN4 (ASC = 0.814) and BLRR (ASC = 0.433), respectively. For MSE, the best and worst predictions were observed in the models DNN_2 (MSE = 2.748) and BLRR (MSE = 11014), respectively. For MAAPE, the best predictions were observed in DNN_1, DNN_2, DNN_3, DNN_4, and PDNN_3, with MAAPE = 0.623, whereas the worst prediction was observed in the BNRR model with MAAPE = 0.738. For ASC, the best model outperformed the worst model by 87.991%, whereas in terms of MSE, the worst model was outperformed by 300.8%, and, for MAAPE, the best model outperformed the worst by 18.460% (Table 3); model abreviations are described in Table 1.

It is very important to highlight that the predictions of each environment were considerably better under the deep learning models (DNN and PDNN) with one, two, three, or four hidden layers when compared with the shallow learning models (GPRR, GPLR, GPER_.75, GPER_.5, GPER_.25, BNRR, and BLRR). However, although there were no statistical differences, the DNN predictions were slightly better than those of the PDNN model. In addition, Bayesian models (with and without the interaction term)] were worse than the classic generalized Poisson models (GPRR, GPLR, GPER_.75, GPER_.5, and GPER_.25). For example, across environments without a genotype ×environment interaction, the average gain in performance of classic generalized Poisson models over the Bayesian methods in terms of MSE was 30.994%, whereas for MAAPE, it was only 3.704%. However, in terms of ASC, the Bayesian models outperformed the classic generalized Poisson models by only 1.16%.

**FIGURE 3:**
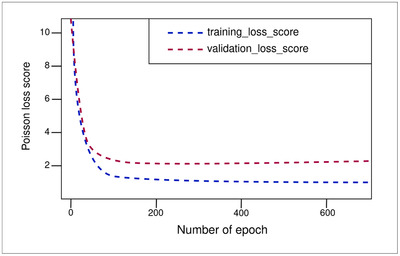
Behavior of the Poisson loss score in the training and validation set increasing the number of epochs. This plot corresponds to the Poisson deep neural network (PDNN) with one hidden layer

**FIGURE 4:**
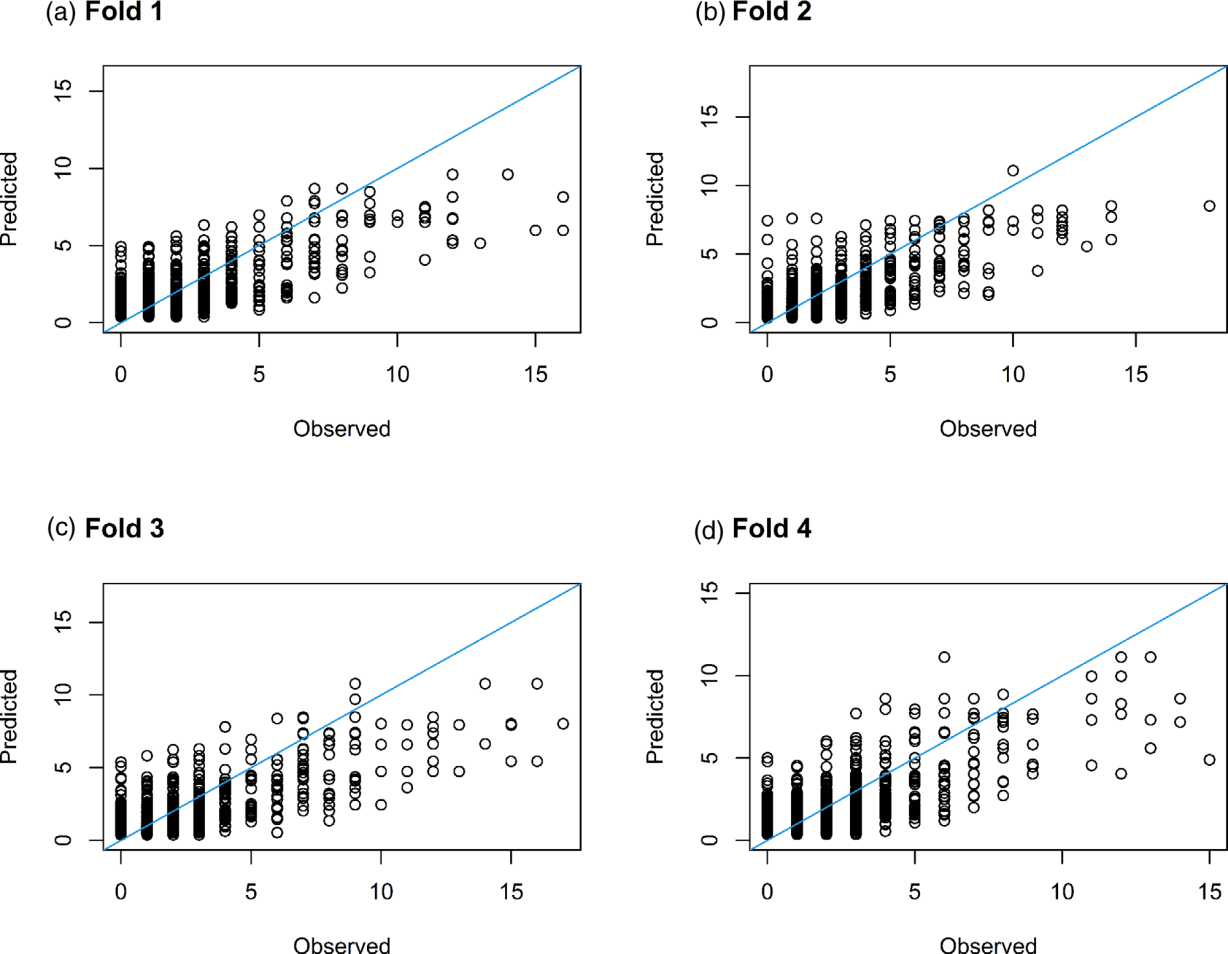
Plot between the predicted vs observed counts for testing sets of (a) Fold 1, (b) Fold 2, (c) Fold 3, and (d) Fold 4 under a Poisson deep neural network (PDDN) with one hidden layer

The results given in Table 3 agree with the comparison for each environment and across environments, where the eight deep learning models (four under DNN and four under PDNN) were superior to all the remaining models, and the worst were the Bayesian models (BNRR and BLRR). Figure 3 illustrates that fewer than 1,000 epochs were enough for the PDNN training process. Figure 4 shows the results of the PDNN model with one hidden layer, for the observed and the predicted values of the corresponding testing set for four out of the five folds (for Folds 1, 2, 3, and 4), which indicates that the predictions have little bias.

### 3.2 Prediction performance taking the genotype×environment interaction into account

We compared the prediction performance of the 15 models across environments and then for each environment, taking the genotype×environment interaction term into account. First, we compared the best and worst prediction models across environments. The PDNN_1 model was the best, with ASC = 0.629, MSE = 1.865, and MAAPE = 0.805. The worst models were BNRR (ASC = 0.584) and BLRR (MSE = 3.472 and MAAPE = 0.8600). The gain in prediction performance in PDNN_1 over the worst model was equal to 7.709% in terms of ASC, equal to 86.217% regarding MSE, and equal to 6.788% for MAAPE. Model abreviations can be found in Table 1.

Next, we compared the prediction performance for each environment. In the environment Batan2012 in terms of ASC and MSE, the best model was PDNN_1 (ASC = 0.537, MSE = 1.435), although for MAAPE, it was GPER_.25 (MAAPE = 0.891). The worst models were BNRR (ASC = 0.513), in terms of ASC, and BLRR (MSE = 1.931, MAAPE = 0.947) for MSE and MAAPE. The best model, PDNN_1, outperformed the worst model by 4.678% in terms of ASC and by 34.564% in terms of MSE, yet the best model GPER_.25 outperformed the worst model by 6.285% in terms of MAAPE (Table 4). In the Batan2014 environment under ASC and MSE, the best model was PDNN_1 (ASC = 0.540, MSE = 1.430), while under MAAPE, it was model GPER_.25 (MAAPE = 0.893). The worst model was BLRR with ASC = 0.509, MSE = 1.937 and MAAPE = 0.931. The gain of the best model over the worst model was 6.090% in terms of ASC, 35.454% in terms of MSE, and 6.271% in terms of MAAPE (Table 4). In the environment Chunchi2014, the best models in terms of ASC were DDN_3 (ASC = 0.814), DNN_4 (ASC = 0.814), PDNN_2 (ASC = 0.814), and PDNN_4 (ASC = 0.814), and the worst model was BNRR (ASC = 0.719). The gain of the best models over the worst models was 13.213%. In terms of MSE, the best model was PDNN_1 (MSE = 2.729) and the worst was BLRR (MSE = 6.549), which means that the gain of the best over the worst model was 139.978%. In terms of MAAPE, the best model was PDNN_1 (MAAPE = 0.618) and the worst was BLRR (MAAPE = 0.684); the gain of PDNN_1 over BLRR was 10.679% (Table 4).

**TABLE 4:** Prediction performance in terms of average Spearman correlation (ASC), mean square error (MSE) and mean arctangent absolute percentage error (MAAPE) considering the genotype×environment (I) interaction of the 15 models; averages of each environment are indicated in bold

Model	Model name^a^	With interaction	Environment	ASC	SE_1^b^	MSE	SE_2	MAAPE	SE_3
1	DNN_1	I	Batan2012	0.533	0.009	1.441	0.042	0.908	0.012
1	DNN_1	I	Batan2014	0.539	0.024	1.437	0.052	0.908	0.011
1	DNN_1	I	Chunchi2014	0.811	0.003	2.751	0.166	0.623	0.013
1	DNN_1	I	**Average**	**0.628**	**0.012**	**1.876**	**0.087**	**0.813**	**0.012**
2	DNN_2	I	Batan2012	0.527	0.011	1.457	0.046	0.91	0.012
2	DNN_2	I	Batan2014	0.535	0.026	1.458	0.054	0.91	0.011
2	DNN_2	I	Chunchi2014	0.813	0.002	2.781	0.164	0.623	0.012
2	DNN_2	I	**Average**	**0.625**	**0.013**	**1.899**	**0.088**	**0.814**	**0.012**
3	DNN_3	I	Batan2012	0.529	0.011	1.452	0.043	0.908	0.012
3	DNN_3	I	Batan2014	0.533	0.026	1.448	0.052	0.909	0.011
3	DNN_3	I	Chunchi2014	0.814	0.001	2.747	0.154	0.621	0.012
3	DNN_3	I	**Average**	**0.625**	**0.013**	**1.882**	**0.083**	**0.813**	**0.012**
4	DNN_4	I	Batan2012	0.527	0.011	1.469	0.044	0.909	0.012
4	DNN_4	I	Batan2014	0.536	0.026	1.463	0.057	0.921	0.005
4	DNN_4	I	Chunchi2014	0.814	0.001	2.792	0.18	0.619	0.013
4	DNN_4	I	**Average**	**0.626**	**0.013**	**1.908**	**0.094**	**0.816**	**0.010**
5	PDNN_1	I	Batan2012	0.537	0.009	1.435	0.042	0.899	0.012
5	PDNN_1	I	Batan2014	0.54	0.015	1.43	0.04	0.899	0.012
5	PDNN_1	I	Chunchi2014	0.809	0.002	2.729	0.155	0.618	0.012
5	PDNN_1	I	**Average**	**0.629**	**0.009**	**1.865**	**0.079**	**0.805**	**0.012**
6	PDNN_2	I	Batan2012	0.527	0.011	1.456	0.045	0.91	0.012
6	PDNN_2	I	Batan2014	0.536	0.026	1.454	0.055	0.91	0.012
6	PDNN_2	I	Chunchi2014	0.814	0.002	2.764	0.161	0.621	0.012
6	PDNN_2	I	**Average**	**0.626**	**0.013**	**1.891**	**0.087**	**0.814**	**0.012**
7	PDNN_3	I	Batan2012	0.5299	0.012	1.4606	0.0402	0.905	0.012
7	PDNN_3	I	Batan2014	0.5347	0.025	1.4588	0.051	0.9043	0.016
7	PDNN_3	I	Chunchi2014	0.8131	0.002	2.8968	0.0851	0.6413	0.003
7	PDNN_3	I	**Average**	**0.626**	**0.013**	**1.939**	**0.059**	**0.817**	**0.010**
8	PDNN_4	I	Batan2012	0.529	0.012	1.471	0.048	0.933	0.012
8	PDNN_4	I	Batan2014	0.535	0.026	1.472	0.053	0.926	0.012
8	PDNN_4	I	Chunchi2014	0.814	0.001	2.919	0.095	0.649	0.012
8	PDNN_4	I	**Average**	**0.626**	**0.013**	**1.954**	**0.065**	**0.836**	**0.012**
9	GPRR	I	Batan2012	0.517	0.006	1.523	0.058	0.894	0.014
9	GPRR	I	Batan2014	0.524	0.016	1.525	0.043	0.894	0.011
9	GPRR	I	Chunchi2014	0.742	0.009	4.337	0.261	0.658	0.01
9	GPRR	I	**Average**	**0.594**	**0.010**	**2.462**	**0.121**	**0.815**	**0.012**
10	GPLR	I	Batan2012	0.522	0.006	1.517	0.055	0.892	0.013
10	GPLR	I	Batan2014	0.525	0.015	1.519	0.044	0.893	0.011
10	GPLR	I	Chunchi2014	0.741	0.009	4.332	0.263	0.66	0.01
10	GPLR	I	**Average**	**0.596**	**0.010**	**2.456**	**0.121**	**0.815**	**0.011**
11	GPER_.25	I	Batan2012	0.52	0.006	1.518	0.056	0.891	0.013
11	GPER_.25	I	Batan2014	0.525	0.015	1.52	0.045	0.893	0.011
11	GPER_.25	I	Chunchi2014	0.741	0.009	4.333	0.263	0.659	0.01
11	GPER_.25	I	**Average**	**0.595**	**0.010**	**2.457**	**0.121**	**0.814**	**0.011**
12	GPER_.5	I	Batan2012	0.522	0.006	1.518	0.055	0.891	0.013
12	GPER_.5	I	Batan2014	0.525	0.015	1.519	0.045	0.893	0.011
12	GPER_.5	I	Chunchi2014	0.741	0.009	4.332	0.263	0.659	0.01
12	GPER_.5	I	**Average**	**0.596**	**0.010**	**2.456**	**0.121**	**0.814**	**0.011**
13	GPER_.75	I	Batan2012	0.522	0.006	1.517	0.055	0.891	0.013
13	GPER_.75	I	Batan2014	0.525	0.015	1.519	0.044	0.893	0.011
13	GPER_.75	I	Chunchi2014	0.741	0.009	4.332	0.263	0.66	0.01
13	GPER_.75	I	**Average**	**0.596**	**0.010**	**2.456**	**0.121**	**0.815**	**0.011**
14	BNRR	I	Batan2012	0.513	0.008	1.531	0.051	0.895	0.013
14	BNRR	I	Batan2014	0.519	0.016	1.533	0.046	0.896	0.011
14	BNRR	I	Chunchi2014	0.719	0.009	4.607	0.277	0.679	0.012
14	BNRR	I	**Average**	**0.584**	**0.011**	**2.557**	**0.125**	**0.823**	**0.012**
15	BLRR	I	Batan2012	0.513	0.009	1.931	0.063	0.947	0.009
15	BLRR	I	Batan2014	0.509	0.019	1.937	0.048	0.949	0.01
15	BLRR	I	Chunchi2014	0.733	0.009	6.549	0.531	0.684	0.007
15	BLRR	I	**Average**	**0.585**	**0.012**	**3.472**	**0.214**	**0.860**	**0.009**

^a^ DNN_1, normal deep neural network with one hidden layer; DNN_2, normal deep neural network with two hidden layers; DNN_3, normal deep neural network with three hidden layers; DNN_4, normal deep neural network with four hidden layers; PDNN_1, Poisson deep neural network with one hidden layer; PDNN_2, Poisson deep neural network with two hidden layers, PDNN_3, Poisson deep neural network with three hidden layers; PDNN_4, Poisson deep neural network with four hidden layers; GPRR, generalized Poisson Ridge regression; GPLR, generalized Poisson Lasso regression; GPER_0.25, generalized Poisson elastic net regression with α = .25; GPER_2, generalized Poisson elastic net regression with α = .5; GPER_.75, generalized Poisson elastic net regression with α = .75; BNRR, Bayseian normal Ridge regression; BLRR, Bayesian log‐normal ridge regression.

^b^ SE_1, SE of ASC; SE_2, SE of MSE; SE_3, SE of MAAPE.

In general, the deep learning models (four under DNN and four under PDNN) were the best in terms of prediction performance, followed by the conventional generalized Poisson regression models (GPRR, GPLR, GPER_.75, GPER_.5, and GPER_.25); the worst models were the Bayesian methods (BNRR and BLRR) (Table 4). For example, for Batan2012, taking the genotype×environment interaction into account, the average of the generalized Poisson regression models was higher than the average of the Bayesian models by 1.559% in terms of ASC, by 13.816% in terms of MSE, and by 3.251% in terms of MAAPE. A similar behavior was observed in the Batan2014 environment, where the generalized Poisson regression models outperformed the average of the Bayesian models by 2.140% in terms of ASC, by 14.474% in terms of MSE, and by 3.247% in terms of MAAPE. Similar gain was observed in Chunchi2014 (the last environment) for the classic generalized Poisson regression models over the Bayesian models. Finally, it is important to point out that the results across environments show significant congruence with the results for each environment, where it is quite clear that the deep learning models (DNN and PDNN) were better than all the other models, followed by the classic generalized Poisson regression models (GPRR, GPLR, GPER_.75, GPER_.5, and GPER_.25), whereas the worst models were the Bayesian normal models (BNRR and BLRR). However, it is important to point out that no significant differences were found between the DNN and PDNN models.

### 3.3 Summary across environments and models for each type of model

**FIGURE 5:**
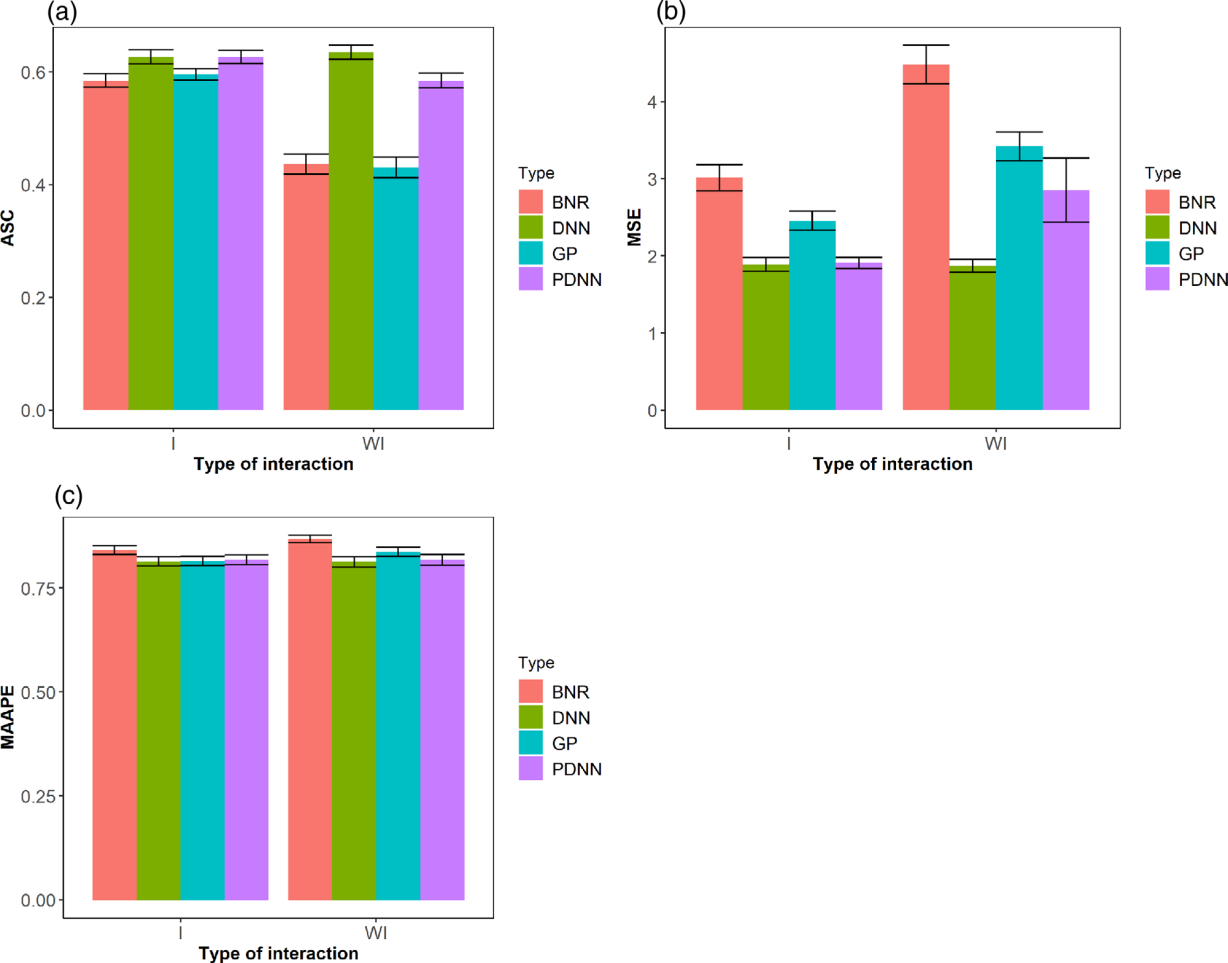
Prediction performance in terms of (a) average Spearman correlation (ASC), (b) mean square error (MSE), and (c) mean arctangent absolute percentage error (MAAPE) across the environments and models of the same type. BNR, Bayesian normal regression; GP, generalized Poisson; DNN, normal deep neural network; PDNN, Poisson deep neural network; I, genotype × environment interaction considered; WI, genotype × environment interaction not considered

The right‐hand panels in Figure 5, without interaction was built by aggregating the results of the 15 models given in Table 3, whereas those on the left‐hand side of Figure 5 (with the interaction) were built by aggregating the results of the 15 models given in Table 4. Under the labels DNN and PDDN, the ASC, MSE, and MAAPE of the four deep learning models of DNN (DNN_1, DNN_2, DNN_3, and DNN_4) and those of the PDNN (PDNN_1, PDNN_2, PDNN_3 and PDNN_4) were averaged (aggregated) across environments. Under the label “generalized Poisson”, the five classic generalized Poisson models (GPRR, GPLR, GPER_.75, GPER_.5, and GPER_.25) were aggregated across environments, whereas under the label “Bayesian normal regression”, the two Bayesian normal methods (BNRR and BLRR) were aggregated across environments. The aggregation was carried out to clearly see the performance of the four types of models (DNN, PDNN, classic Poisson regression, and Bayesian normal models).

Figure 5 indicates that Bayesian models (BNRR and BLRR) with genotype ×environment interaction were better than those in which the genotype × environment interaction was ignored, by 33.944% in terms of ASC, by 48.837% in terms of MSE, and by 3.088% in terms of MAAPE. Moreover, classic generalized Poisson regression models were better when they consider the genotype × environment interaction by 38.283% in terms of ASC, by 39.024% in terms of MSE and by 2.699% in terms of MAAPE. However, under DNN, no relevant differences were observed in the three metrics (ASC, MSE, and MAAPE) between considering the genotype×environment interaction and ignoring it. Under the PDNN models, we observed no differences in terms of prediction performance with and without the genotype ×environment interaction; however, in terms of the ASC and MSE, when the genotype ×environment interaction was considered, the prediction performance was better by 7.363 and 49.214%, respectively, than when the interaction term was ignored.

Additionally, Figure 5a clearly shows that the best performance in terms of ASC was demonstrated by the deep learning models (DNN and PDNN), whereas the worst was by the Bayesian and classic generalized Poisson models. However, Figure 5 also shows that without the genotype×environment interaction, the DNN models were better than the PDNN models, but if the interaction was considered, no differences were observed between these deep learning models. Across models and environments, the PDNN model outperformed the GP model by 5.20% (in terms of ASC) with the interaction term and 35.498% (in terms of ASC) without it. Regarding the BNRR model, the PDNN model was superior by 7.363% (in terms of ASC) with the interaction term and by 33.944% (in terms of ASC) without it. The same behavior is shown in Figure 5b, where we can see that the deep learning models were the best, although without the genotype ×environment interaction, the DNN models were slightly better than the PDNN models. However, Figure 5c shows no relevant differences among the classic generalized Poisson, DNN, and PDNN models with and without the genotype ×environment interaction, and these models were slightly better than the Bayesian methods (BNRR and BLRR).

## 4 DISCUSSION

In this study, an application of the PDNN for count data proposed by Montesinos‐López et al. (2020) was implemented. The PDNN is novel, since it can be implemented under the umbrella of deep learning, allowing us to capture nonlinear patterns in the data that cannot be captured with current generalized linear models for count data (Bayesian or classic). The ability of the Poisson deep learning model to capture complex patterns can be attributed to the fact that it is able to incorporate more than one hidden layer. Hidden layers perform nonlinear transformations (such as RELU, sigmoid, exponential, etc.) of the inputs and outputs of hidden neurons. When the patterns are very complex, more than one hidden layer is required in most cases (Chollet & Allaire, 2017). Because this model can be implemented with Keras as the front‐end and Tensorflow as the back‐end, it is possible to implement various hidden layers in a very manageable framework, allowing us to use different activation functions (RELU, leaky RELU, etc.) to capture the nonlinear patterns in the hidden layers. Furthermore, this model can be used for moderate and large datasets because the training process is performed with batches of the whole training set; in addition, it allows the use of graphics processing unit computing, thus avoiding memory problems.

No great differences were observed between the DNN and PDNN models with or without the interaction term, which can be partially attributed to the fact that the hidden layers capture nonlinear patterns and interaction terms, and it is expected that the larger the number of hidden layers, the more efficient the deep neural network will be in capturing these patterns. For this reason, including the interaction term as an input of deep learning algorithms is not often required; this pattern has been observed in some publications of deep learning applied for GS (Montesinos‐López, Montesinos‐López, Gianola, et al., 2018, Montesinos‐López, Montesinos‐López, Crossa, et al. 2018; Montesinos‐López et al., 2019a, 2019b). Not explicitly including information about the genotype ×environment interaction as an input of deep learning models has the advantage that fewer computational resources are needed for their implementation, since the dimensionality of the input is lower.

The PDNN proposed by Montesinos‐López et al. (2020) should be preferred over any machine learning model that assumes that the response variable is continuous because the PDNN was built under the assumption that the response variable is Poisson‐distributed, guaranteeing that all predictions are non‐negative (which is not guaranteed by any machine learning model that assumes that the response variable is continuous) (Montesinos‐López et al., 2015; Montesinos‐López et al., 2016; Montesinos‐López et al., 2017). When other machine learning methods are used (assuming a continuous outcome), negative outputs must be truncated to zero, and it is unclear how this affects the optimality of the predictive distribution (Montesinos‐López et al., 2015; Montesinos‐López et al., 2016; Montesinos‐López et al., 2017). However, in terms of prediction performance there is also evidence that many times (for particular datasets), specific machine learning methods that assume a continuous response variable give a similar prediction performance to those models that assume that the response variable is count data. However, the models with the right assumption about the response variable always guarantee a good performance, even when, in some circumstances, they do not outperform conventional models for continuous response variables.

The prediction performances of DNN and PDNN were very competitive (and, in many cases, better) with the classic GS models, although the count dataset was not large, since it was composed of 6,480 observations (115 lines, 3 environments, and around 56.348 observations per line). Our results show that sometimes, even with small datasets, it is possible to obtain a reasonable prediction performance. However, empirical evidence shows that deep learning models are more efficient in the context of large datasets (with at least 25,000 observations) (Emmert‐Streib et al., 2020). For this reason, our results are very attractive, because although our dataset cannot be classified as large, the performance of the proposed PDNN was competitive (but not better than that of the DNN) with the classic GS models; for this reason, we encourage more research to support our findings.

Although there are many advantages to using the PDNN model, there are also some drawbacks, similar to those of any deep learning model, as many hyperparameters need to be tuned to successfully implement the proposed Poisson deep learning model. In this vein, the tuning process is both time‐consuming and challenging. However, even with this limitation, the PDNN model can be implemented quite easily, even by users without a strong background in statistics and computer science. For these reasons, we are confident that this is a powerful tool for GS with count traits as response variables, since it does not have the deficiencies of Bayesian Poisson regression models, which are slow at generating samples with analytical or numerical Gibbs samplers and can only capture linear patterns.

As mentioned above, in order to successfully implement deep learning methods, the tuning process should be done efficiently. This task is difficult, since many hyperparameters (percentage of dropout, number of units in each layer, activation functions, learning rate, number of epochs, etc.) need to be tuned in deep learning models. However, the tuning process is more of an art than a science because there is not a unique and universal method that provides the best combination of hyperparameters that guarantee good prediction performance in the testing set. For this reason, it is important to point out that our results are limited to the grid values used for the following hyperparameters: units, the λ hyperparameter for Lasso regularization, number of epochs, dropout percentage, batch size, validation split, learning rate, number of hidden layers, activation functions, and type of architecture. All hyperparameters are important, but some carry out similar tasks. For example, the percentage of dropout is important to maintain stability in weight estimates and avoid overfitting, but this task can also be done with methods of regularization like Ridge regularization, Lasso regularization, elastic net regularization, and the early stopping method. However, it is important to continue improving the tools for tuning hyperparameters under more automatic frameworks like Keras‐tuner and auto‐Keras, which will help in simplifying this complex task.

Regarding the time of implementation, DNN models require many computational resources. As pointed out above, with the use of graphics processing units (with thousands of cores), it is possible to perform trillions of operations per second, allowing us to significantly reduce the implementation time of the DNN model. However, graphics processing units with thousands of cores are still unavailable for many users, and programing should be efficient to be able to perform the operations much faster than with a conventional central processing unit.

Our results did not indicate that the PDNN model was better than the DNN model, but in general, it was better than the classic generalized Poisson regression and Bayesian regression methods. In theory, this was expected, since the implemented PDNN model assumes that the response variable has a Poisson distribution and can capture nonlinear patterns through hidden layers that then execute nonlinear activation functions. The adequate performance in many scenarios of the PDNN model over the generalized Poisson regression models (GPRR, GPLR, GPER_.75, GPER_.5, and GPER_.25) is attributed to the fact that the PDNN model can capture nonlinear patterns, whereas Poisson regression is only able to capture linear patterns, even when the models also assume a Poisson distribution in the response variable. However, it is important to point out that our conclusions are not generalizable because they are only valid for this dataset. Although the proposed PDNN model is an alternative tool that breeders can explore for GS in the context of count traits, it can enrich the analytical tools available for genomic prediction and can produce better predictions for count data with complex nonlinearities. Regarding the time implementation of the proposed methods, those which took less time were the generalized Poisson regression models, followed by the Bayesian methods, whereas the worst were the deep learning methods (DNN and PDNN). For this reason, it is frequently more practical and easier to use conventional methods for small datasets with less complex patterns in the inputs.

It is also important to point out that in the context of excess zeros in the response variable, the PDNN should not be the best option. For this reason, Montesinos‐López et al. (2021) proposed a model for this specific context: Count data with excess zeros can also be used in the context of a large number of independent variables and a small sample size, as well as for moderate or large datasets. However, this model was developed under a random forest framework.

The different performance of the Bayesian normal models (BNRR, BLRR) and the generalized Poisson regression models (GPRR, GPLR, GPER_.75, GPER_.5, and GPER_.25) can be attributed to the following issues: (a) the generalized Poisson regression models use a part of the training set to estimate the parameter of regularization (λ); (b) the generalized Poisson regression models do not use weak priors for the beta coefficients, like the Bayesian normal models do; (c) the generalized Poisson regression models only use one parameter of regularization (λ) for all the input information (environments, genotypes, and genotype×environment interaction); and (d) the generalized Poisson regression models were trained via gradient descendent methods, whereas the Bayesian normal models were trained with the Gibbs sampler.

The model for count data used in this study (PDNN) could be used in other areas of research such as biomedical informatics, where reviewed studies have shown the need for a greater use of count data models, including Poisson regression and negative binomial methods, among others. Du et al. (2011) reviewed the impact of health information technology and advocated the use of more effective statistical models to better account for the characteristics of count data and not rely on suboptimal statistical models, such as ordinary least squares.

Finally, even though the implemented PDNN model was evaluated with only one dataset, the results indicate that it is competitive with DNN, as well as with conventional statistical learning tools, with the advantage that it can be implemented in existing software (such as Keras as the front‐end and Tensorflow as the back‐end) that is very user‐friendly. However, we expect the deep learning models (DNN and PDNN) will perform better with larger datasets, since there is considerable evidence to support this. As such, we encourage other researchers to implement the PDNN model with other count datasets to increase the body of evidence indicating that it performs better than statistical machine learning algorithms for large datasets.

## 5 CONCLUSIONS

In this paper, we applied a deep learning model for count traits. This Poisson deep learning model was evaluated on a real dataset that was benchmarked with deep neural networks for continuous outcomes (DNN) and conventional statistical learning methods (generalized Poisson regression and Bayesian linear regression). The results show that the Poisson deep learning model is competitive with the conventional deep learning methods and statistical learning methods. As such, the deep learning model is a novel and attractive analytical tool that can be used in GS for count traits as it can capture nonlinear patterns through the implementation of more than one hidden layer, which is not possible with conventional generalized Poisson regression, which only captures linear patterns. Finally, the PDNN model addresses the lack of efficient genomic prediction models for count data in the context of moderate or large datasets that contain more independent variables than observations.

## CONFLICT OF INTEREST

The authors declare no conflict of interest.

## AUTHOR CONTRIBUTIONS

Jose Crossa: conceptualization, investigation, methodology. Osval Montesinos‐Lopez: conceptualization, formal analysis, investigation, methodology. Jose C. Montesinos‐Lopez: conceptualization, investigation, methodology. Jose Alberto Barron: conceptualization, formal analysis, methodology. Eduardo Salazar: conceptualization, methodology. Abelardo Montesinos‐Lopez: conceptualization, formal analysis, investigation, methodology. Raymundo Buenrostro‐Mariscal: conceptualization, formal analysis.
